# Soil Bacteria in Urban Community Gardens Have the Potential to Disseminate Antimicrobial Resistance Through Horizontal Gene Transfer

**DOI:** 10.3389/fmicb.2021.771707

**Published:** 2021-11-23

**Authors:** Abdullah Ibn Mafiz, Yingshu He, Wei Zhang, Yifan Zhang

**Affiliations:** ^1^Department of Nutrition and Food Science, Wayne State University, Detroit, MI, United States; ^2^Department of Human Sciences, Tennessee State University, Nashville, TN, United States; ^3^Department of Food Science and Nutrition, Illinois Institute of Technology, Chicago, IL, United States; ^4^Center for Food Safety, University of Georgia, Griffin, GA, United States

**Keywords:** antimicrobial resistance, soil bacteria, horizontal gene transfer, urban agriculture, whole genome sequencing

## Abstract

Fifteen soil and 45 vegetable samples from Detroit community gardens were analyzed for potential antimicrobial resistance contamination. Soil bacteria were isolated and tested by antimicrobial susceptibility profiling, horizontal gene transfer, and whole-genome sequencing. High-throughput 16S rRNA sequencing analysis was conducted on collected soil samples to determine the total bacterial composition. Of 226 bacterial isolates recovered, 54 were from soil and 172 from vegetables. A high minimal inhibitory concentration (MIC) was defined as the MIC greater than or equal to the resistance breakpoint of *Escherichia coli* for Gram-negative bacteria or *Staphylococcus aureus* for Gram-positive bacteria. The high MIC was observed in 63.4 and 69.8% of Gram-negative isolates from soil and vegetables, respectively, against amoxicillin/clavulanic acid, as well as 97.5 and 82.7% against ampicillin, 97.6 and 90.7% against ceftriaxone, 85.4 and 81.3% against cefoxitin, 65.8 and 70.5% against chloramphenicol, and 80.5 and 59.7% against ciprofloxacin. All Gram-positive bacteria showed a high MIC to gentamicin, kanamycin, and penicillin. Forty of 57 isolates carrying *tetM* (70.2%) successfully transferred tetracycline resistance to a susceptible recipient via conjugation. Whole-genome sequencing analysis identified a wide array of antimicrobial resistance genes (ARGs), including those encoding AdeIJK, Mex, and SmeDEF efflux pumps, suggesting a high potential of the isolates to become antimicrobial resistant, despite some inconsistency between the gene profile and the resistance phenotype. In conclusion, soil bacteria in urban community gardens can serve as a reservoir of antimicrobial resistance with the potential to transfer to clinically important pathogens, resulting in food safety and public health concerns.

## Introduction

The environmental reservoir of antimicrobial resistance has drawn extensive research attention due to their connection with antimicrobial resistance in human pathogens (Hu et al., 2018; Kraemer et al., 2019; Zhang et al., 2019b; Scott et al., 2020). The high DNA sequence similarity of environmental antimicrobial resistance genes (ARGs) with human pathogens (Forsberg et al., 2012) and fecal microorganisms (Nesme et al., 2014) suggests a common pool of ARG between human and the environment. Of the many routes through which human acquires ARG from the environment, agriculture plays a key role since agricultural soil not only harbors a large natural resistome but also receives antimicrobial resistance from human and animal wastes, which can return to human and animals through agricultural products and practices (Tiedje et al., 2019). Although multidrug-resistant bacteria of diverse genera have been found in urban, agricultural, and pristine soils (Walsh and Duffy, 2013), the food safety implication of the potential of soil bacteria in disseminating ARGs remains under investigated.

A previous research investigating manure-amended soil and lettuce cultivated in the soil found that rhizosphere soil on lettuce showed more diverse ARGs than other parts of the lettuce, suggesting that lettuce may be able to acquire ARGs from soil resistome (Zhang et al., 2019a). Peri-urban arable soils demonstrated more diverse and abundant ARGs compared to peri-urban pristine soil, implicating possible food safety concern associated with vegetables grown in these soils (Xiang et al., 2018). According to the limited research performed in urban agriculture, soil bacteria recovered from urban community gardens were commonly resistant to ampicillin, cefoxitin, ceftriaxone, chloramphenicol, gentamicin, kanamycin, and penicillin (Mafiz et al., 2018). Metagenomic data also revealed the prevalence and abundance of genes encoding resistance to quinolones, β-lactams and tetracyclines in soil. Another study recovered *E. coli* and *Enterococcus* from vegetables grown in urban gardens and found that more than 60% of *E. coli* were ampicillin resistant as well as 86.8% of Enterococcal isolates resistant to ampicillin, ciprofloxacin, erythromycin, streptomycin, and tetracycline (Perera et al., 2020).

The correlation between mobile genetic elements (MGE) and the occurrence of ARG has been reported in many studies and demonstrated as indirect evidence of horizontal gene transfer in agricultural environment. A recent study in China detected 33 ARGs in agricultural soils from eight sampling sites based on urban distribution pattern and irrigation channel network (Shi et al., 2020). According to the study, agricultural sites closer to urban areas had significantly higher antimicrobial-resistant bacteria and ARGs compared to the remote sites. A significant correlation between *intI-*1 and ARGs (*aad*A1, *aad*A2, *mph*A, *sul*I, and *sul*II) observed in the study indicates a potential role of horizontal gene transfer in ARG dissemination in agricultural soil. ARG and MGE correlation has also been reported in archived urban and suburban soils (Zhao et al., 2019). Zhao et al. detected 164 ARGs conferring resistance to all major antibiotics used in humans and animals, including aminoglycosides, beta-lactams, chloramphenicols, sulfonamides, and vancomycin. *intI*-1 was reported as the predominant MGE (Zhao et al., 2019).

Despite substantial research on antimicrobial resistance in agricultural environment, limited knowledge is available in urban agricultural environment, especially at isolate and molecular level. Importantly, it remains unknown as to the potential of antimicrobial resistance transfer from soil microorganisms to bacteria of food safety significance. Therefore, this study aimed to determine the role of urban community gardens as a potential reservoir and dissemination route of antimicrobial-resistant bacteria and ARGs.

## Materials and Methods

### Sample Collection

Soil and vegetables were collected from three gardens (E, G, and O) located in the metro Detroit area. Gardens E and G were located near the city center while garden O was in an outer suburban area. Agricultural practices on the garden plots are provided in Supplementary Document 1. Fifteen soil samples (5 from each garden) and 45 vegetables (21 from E, 5 from G, and 19 from O) were collected. The five soil samples from each garden were spread out in a 20 m × 20 m plot and collected at a depth of 0–7.5 cm. Each sample of 1 kg was pooled by five subsamples collected approximately 10 cm from each other at the center and four corners around the same sampling spot. Vegetables were based on the availability during the collection time and included root vegetables and leafy greens. Samples were collected in clean Ziploc bags and transported using an ice-filled cooler to the lab within 2 h.

### Isolation and Identification of Soil Bacteria

Antimicrobial-resistant bacteria were isolated and identified by following a previous protocol (Mafiz et al., 2018). Briefly, 50 g of soil or vegetable sample was mixed with 450 ml of brain heart infusion (BHI) (Difco, Sparks, MD, United States) and homogenized, followed by streaking on R2A agar (Difco) containing 20 μg/mL of ampicillin, streptomycin, or tetracycline. Agar plates were incubated at 30°C for 4 to 5 days and up to five colonies of different morphology were picked for purification. Bacteria DNA was extracted using a boiling method, followed by PCR amplification of the 16S rRNA gene. PCR reactions of 25 μl included 5 μl of template DNA, 0.5 μM of each primer, 1 × PCR buffer, 4.0 mM of MgCl_2_, 200 μM of each dNTP, and 1U *Taq* DNA polymerase (Promega, Madison, WI, United States). PCR products were sent to Eton Bioscience Laboratories, NJ for purification and sequencing. Resulted DNA sequences were analyzed using the Ribosomal Database Project (RDP) website^1^.

### Antimicrobial Susceptibility Testing

The Minimum Inhibitory Concentration (MIC) of the isolates was measured using the Gram-positive and Gram-negative Sensititre plates from the Sensititre Antimicrobial Susceptibility System (Trek Diagnostic Systems, Westlake, OH, United States). The resistance breakpoints of *E. coli* ATCC 25922 and *S. aureus* ATCC 29213 according to the Clinical and Laboratory Standards Institute (CLSI) were used to interpret the MICs of Gram-negative and Gram-positive bacteria, respectively. A high MIC was defined as the MIC higher than or equal to the resistance breakpoint of the reference strain.

### Determination of Soil Microbial Composition

Microbial composition in soil was determined by high-throughput 16S rRNA sequencing. Soil DNA was extracted using the DNeasy PowerSoil kit (Qiagen, Valencia, CA, United States) according to the manufacturer’s instructions. After checking the quality and quantity of the extracted DNA, a 16S rDNA sequencing library was constructed using the 16S Metagenomic Sequencing Library Preparation protocol (Illumina, San Diego, CA, United States) modified by Dr. Karen Jarvis from FDA. Briefly, Omni Klentaq PCR Kit (DNA Polymerase Technology, St. Louis, MO, United States) was used for initial PCR using locus-specific primers to amplify the V1-V3 hyper-variable region of the bacterial 16S rRNA gene (Chakravorty et al., 2007; Lusk et al., 2012; Klindworth et al., 2013; Yarza et al., 2014). The Illumina sequencing adapters and dual-index barcodes were added to the purified PCR products using Nextera XT Index Kit (Illumina) by a limited cycle PCR. Prepared libraries were sequenced on the MiSeq sequencing platform (Illumina) using paired 300-bp reads and MiSeq v3 reagents, following standard Illumina sequencing protocols. Data were analyzed using the Metagenomics workflow to perform a taxonomic classification against the Greengenes database.

### Conjugation Experiment

The conjugation experiment was conducted to demonstrate the horizontal transfer of tetracycline resistance from soil bacteria to *E. coli* or *Enterococcus*. Bacteria of an MIC ≥ 16 μg/ml of tetracycline were selected as donors for conjugation. All donor strains were *tet*M positive as identified by PCR. *E. coli* DH5α (amp^r^, kan^r^) and *Enterococcus faecalis* JH2-2 (rif^r^, fus^r^) were used as recipient strains for Gram-negative and Gram-positive isolates, respectively. Conjugation was performed by the filter mating method (Agersø et al., 2006) with modifications. Briefly, overnight culture of the donor strain was grown in BHI (Difco) containing tetracycline (15 μg/ml). The recipients *E. coli* DH5α and *Enterococcus faecalis* JH2-2 were grown in BHI (Difco) containing 50 μg/ml of kanamycin and rifampicin, respectively. The mating mixture with a donor-to-recipient ratio of 1:1 was then placed on a 0.45-μm-pore-size filter and incubated on BHI agar (Difco) at 30°C for 2 days. The filter was washed and vortex-mixed in BHI broth. The mating mixture was spread onto BHI agar containing tetracycline (15 μg/mL) and kanamycin (50 μg/ml) for Gram-negative bacteria and tetracycline (15 μg/mL) and rifampicin (50 μg/ml) for Gram-positive bacteria. Transconjugants were confirmed by PCR detection of *tet*M.

### Whole-Genome Sequencing

A total of 23 isolates, seven or eight from each garden, based on the diversity of antimicrobial susceptibility profiles, were selected for whole-genome sequencing. Genomic DNA was extracted, followed by the preparation of paired-end libraries using a Nextera DNA Flex Library Prep Kit (Illumina, San Diego, CA, United States). Pooled library was diluted and denatured before being loaded into a MiSeq reagent cartridge version 3 (Illumina, San Diego, CA, United States). Sequencing by synthesis of the paired-end 300 bp reads was conducted using an on-site MiSeq platform (Illumina, San Diego, CA, United States). FastQC.0.11.6^2^ was used to analyze the quality of the read data. Trimmomatic 0.36^3^ (Bolger et al., 2014) was employed to remove the adaptors and low-quality bases from the reads. The trimmed reads were assembled using the *de novo* assembler SPAdes 3.11.1^4^ (Bankevich et al., 2012), using default parameters with a broad range of k-mer values (from 21 to 127). Genome assembly quality was assessed by QUAST version 5.0.2 (Supplementary Table 1). Genome size was estimated by KmerGenie (Chikhi and Medvedev, 2014). All statistics were based on contigs of size ≥ 500 bp (Mikheenko et al., 2018). The assembled contigs for each genome were used as queries against the Comprehensive Antibiotic Resistance Database (CARD^5^) to identify ARGs (McArthur et al., 2013).

### Statistical Analysis

Chi-square tests were conducted to compare the MIC distribution of isolates between soil and vegetables using SPSS v. 21.0 (IBM SPSS, Chicago, IL, United States). *P* < 0.05 is statistical significance.

## Results

### Isolation and Identification of Antimicrobial-Resistant Bacteria From Soil and Vegetables

**FIGURE 1:**
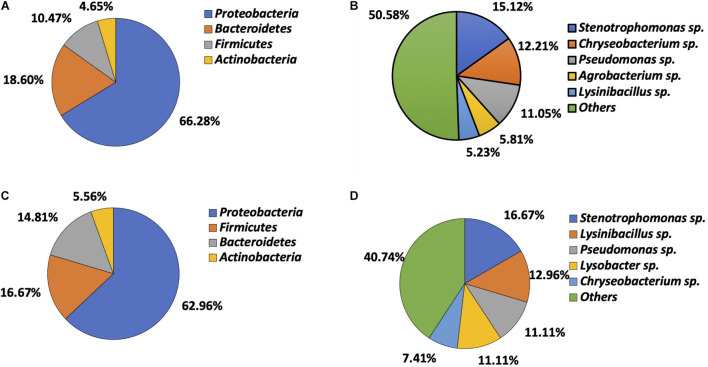
Microbial composition determined by bacteria identification. **(A)** Phylum distribution in vegetable bacteria (*n* = 172). **(B)** Genus distribution in vegetable bacteria (*n* = 172). **(C)** Phylum distribution in soil bacteria (*n* = 54). **(D)** Genus distribution in soil bacteria (*n* = 54).

A total of 226 bacteria were isolated, including 54 from soil (13 Gram-positive and 41 Gram-negative) and 172 from vegetables (33 Gram-positive and 139 Gram-negative). Gram-negative bacteria (*n* = 180) were predominant. Bacteria from vegetables belonged to four phyla, including *Proteobacteria* (*n* = 114, 66.28%), *Bacteroidetes* (*n* = 32, 18.60%), *Firmicutes* (*n* = 18, 10.47%), and *Actinobacteria* (*n* = 8, 4.65%) (Figure 1A). Twenty-nine genera were identified, *Stenotrophomonas* (*n* = 26, 15.12%), *Chryseobacterium* (*n* = 21, 12.21%), *Pseudomonas* (*n* = 19, 11.05%), *Agrobacterium* (*n* = 10, 5.81%), and *Lysinibacillus* (*n* = 9, 5.23%) being most prevalent. The remaining genera combined accounted for 50.58% (*n* = 87) of total isolates (Figure 1B). Isolates recovered from soil also belonged to the same four phyla, with *Proteobacteria* being predominant (*n* = 34, 62.96%), followed by *Firmicutes* (*n* = 9, 16.67%), *Bacteroidetes* (*n* = 8, 14.81%), and *Actinobacteria* (*n* = 3, 5.56%) (Figure 1C). They consisted of 21 genera. *Stenotrophomonas* (*n* = 9, 16.67%), *Lysinibacillus* (*n* = 7, 12.96%), *Pseudomonas* (*n* = 6, 11.11%), *Lysobacter* (*n* = 6, 11.11%), and *Chryseobacterium* (*n* = 4, 7.41%) were most common. The remaining genera combined made up 40.74% of the isolates (*n* = 22) (Figure 1D).

### Antimicrobial Susceptibility Profile of Bacteria

**FIGURE 2:**
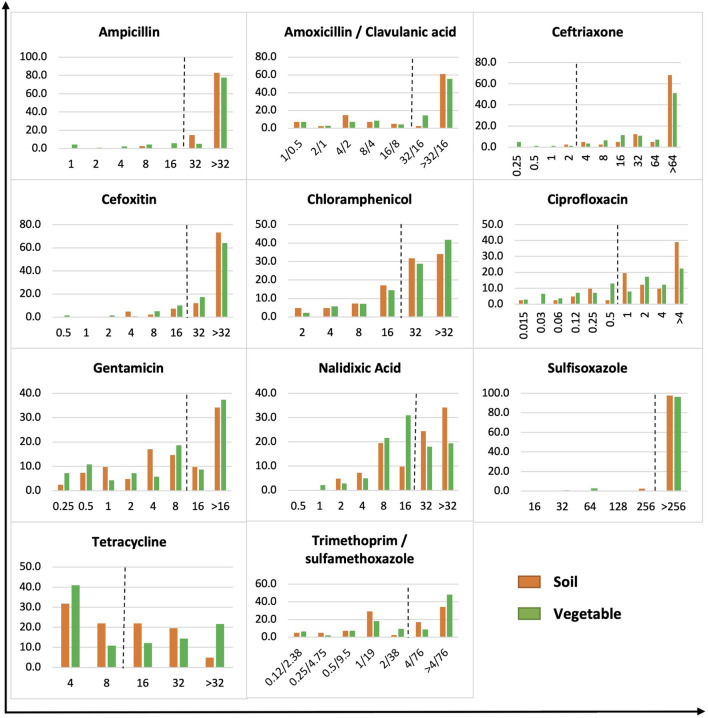
MIC distribution of gram-negative bacteria from soil and vegetable origin. *X*-axis is MIC in μg/ml. *Y*-axis is % of soil or vegetable isolates. The dot line on each figure stands for the breakpoint of *E. coli* against the specific antibiotic.

**FIGURE 3:**
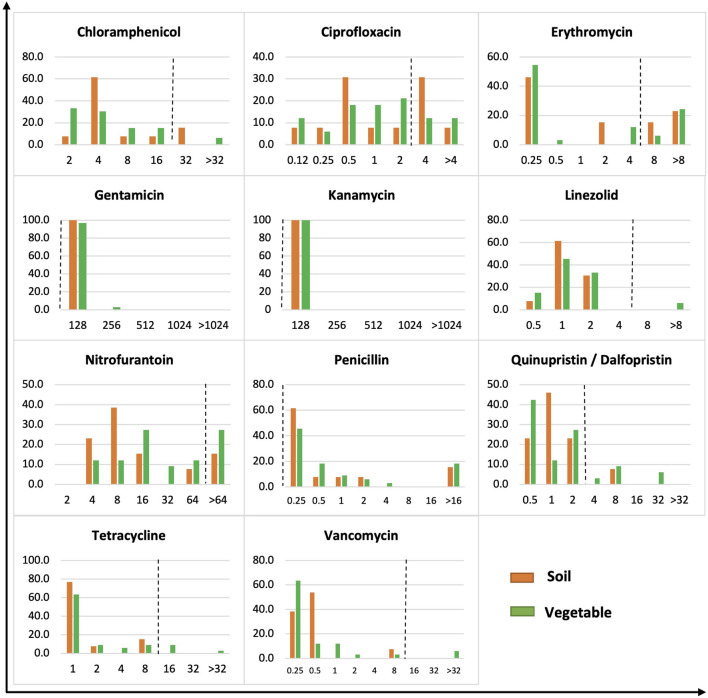
MIC distribution of gram-positive bacteria from soil and vegetable origin. *X*-axis is MIC in μg/ml. *Y*-axis is % of isolates of soil or vegetable isolates. The dot line on each figure stands for the breakpoint of *S. aureus* against the specific antibiotic.

The MICs of Gram-negative bacteria were skewed toward higher antimicrobial concentrations, whereas a more even distribution was observed on Gram-positive bacteria (Figures 2, 3). Of 180 Gram-negative bacteria isolated, the majority showed a high MIC to most antibiotics tested regardless of origin (*p* > 0.05) (Figure 2). For example, bacteria with a high MIC to amoxicillin/clavulanic acid were 63.4 and 69.8% in soil and vegetable isolates, respectively, compared to 97.6 and 90.7% for ceftriaxone, 85.4 and 81.3% for cefoxitin, 65.8 and 70.5% for chloramphenicol, 43.9 and 46.0% for gentamicin, 97.6 and 96.4% for sulfisoxazole, 46.4 and 48.2% for tetracycline, and 51.2 and 56.8% for trimethoprim/sulfamethoxazole. Significant difference was observed between soil and vegetable isolates in the profile for ampicillin, ciprofloxacin, and nalidixic acid (*p* < 0.05). Isolates with a high MIC to ampicillin were demonstrated by 97.5 versus 82.7% of bacteria from soil and vegetable samples, respectively. The prevalence was 80.5% (soil) and 59.7% (vegetable) for ciprofloxacin, and 58.5% (soil) and 37.4% (vegetables) for nalidixic acid.

The 46 Gram-positive bacteria showed a wide distribution of MICs across the tested concentrations of antibiotics (Figure 3). All Gram-positive bacteria, regardless of soil or vegetable origin, showed an MIC of 128 μg/ml to gentamicin and kanamycin compared to the *S. aureus* resistance breakpoint of 16 μg/ml. Similarly, all isolates demonstrated a high MIC for penicillin. Bacteria showing a high MIC for chloramphenicol had a prevalence of 15.4 and 6.1% in soil and vegetables, respectively, compared to 38.5 and 24.2% for ciprofloxacin, 38.5 and 30.3% for erythromycin, 15.4 and 27.3% for nitrofurantoin, and 7.7 and 18.2% for quinupristin/dalfopristin. High MIC against linezolid, tetracycline, and vancomycin was observed in 6.1, 12.1, and 6.1% of isolates, respectively, of vegetable origin, whereas no isolates from soil demonstrated an MIC greater than or equal to the *S. aureus* resistance breakpoint for the corresponding antibiotics.

### Bacteria Diversity in Soil Revealed by High-Throughput 16S rRNA Sequencing

**FIGURE 4:**
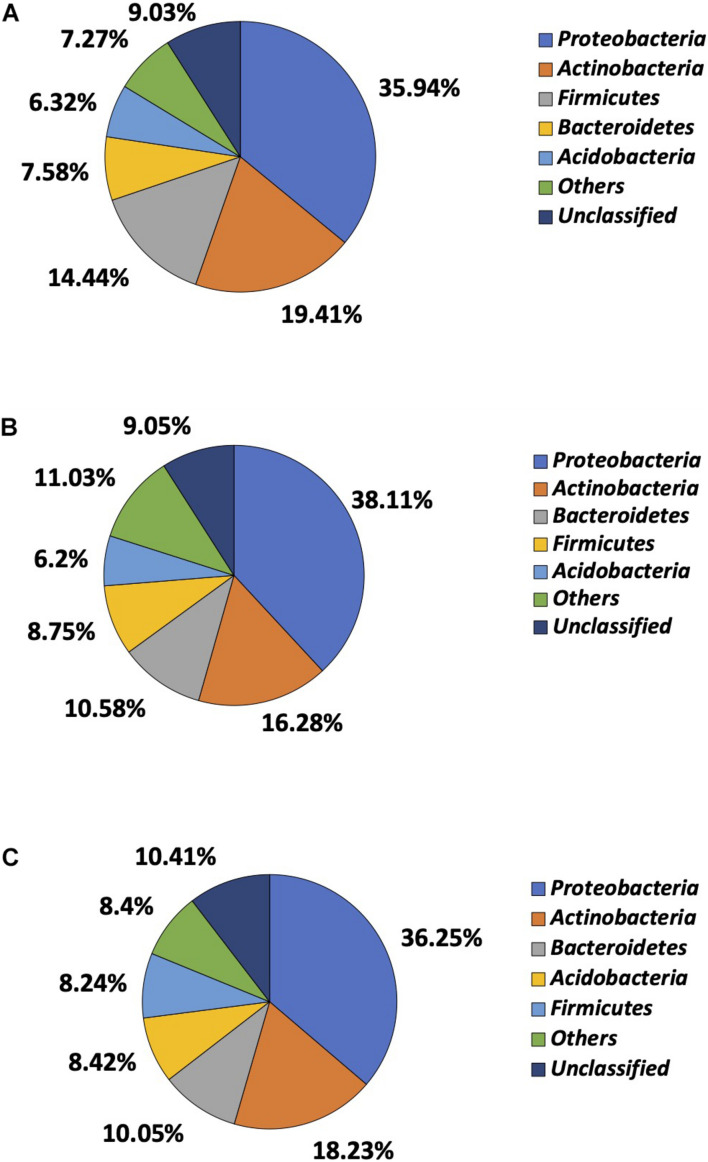
Microbial composition determined at phylum level by 16S rRNA sequencing. **(A)** Phylum distribution of soil bacteria in Garden E. **(B)** Phylum distribution of soil bacteria in Garden G. **(C)** Phylum distribution of soil bacteria in Garden O.

Of more than 30 phyla identified by 16S rRNA sequencing, *Proteobacteria*, *Actinobacteria*, *Firmicutes*, *Bacteroidetes*, and *Acidobacteria* were most prevalent, though the order of prevalence varied by garden (Figure 4). *Proteobacteria* were most common in garden E and identified in 35.94% of the reads, followed by *Actinobacteria* (19.41%), *Firmicutes* (14.44%), *Bacteroidetes* (7.58%), and *Acidobacteria* (6.32%). The 16S rRNA sequencing failed to classify 9.03% of the reads to the phylum level in garden E (Figure 4A). The top five phyla identified in Garden G were comprised of 79.92% of the total reads, with *Proteobacteria* being most prevalent and identified in 38.11% of the reads, followed by *Actinobacteria* (16.28%), *Bacteroidetes* (10.58%), *Firmicutes* (8.75%), and *Acidobacteria* (6.20%) (Figure 4B). Top five phyla identified in Garden O were *Proteobacteria* (36.25%), *Actinobacteria* (18.23%), *Bacteroidetes* (10.05%), *Acidobacteria* (8.42%), and *Firmicutes* (8.24%) (Figure 4C). When comparing the cultured and 16S rRNA sequencing methods, except for *Proteobacteria* that predominated as revealed by both methods, the ranking of other phyla differed between the two methods.

### Conjugation

A total of 55 Gram-negative and 2 Gram-positive bacteria with a high MIC to tetracycline were tested for conjugation. Forty of 57 bacteria (70.2%) were able to make the recipient tetracycline resistant after conjugation (Table 1). The successful transfer of tetracycline resistance was confirmed by *tetM* presence in all 40 transconjugants. Out of 55 Gram-negative isolates tested, 28 were *Stenotrophomonas*, followed by 17 *Chryseobacterium*, five *Sphingobacterium*, and one each of *Dyadobacter*, *Lysobacter*, *Pseudomonas*, *Agrobacterium*, and *Variovorax*. Approximately 72% (20/28) of *Stenotrophomonas* successfully transferred tetracycline resistance to the recipient at a conjugation frequency of 2.28 × 10^–4^ to 3.36 × 10^–3^/recipient cell. Conjugation was successful in 58.8% (10/17) of *Chryseobacterium* and 80% (four of five) of *Sphingobacterium*. The only two Gram-positive bacteria tested, *Microbacterium* and *Curtobacterium*, were positive for conjugation. No distinction was observed in conjugation frequency among gardens or between soil and vegetable isolates. However, more soil isolates from Garden E were positive for conjugation, which is consistent with a higher number of bacteria with high MIC to tetracycline observed in Garden E soils. This could be due to special soil factors in the garden that may select certain antibiotic resistance and will require further research.

**TABLE 1:** Conjugation results of 40 soil and vegetable isolates from three gardens.

Garden	Source	Donor (Number of isolates)	Conjugation rate (range)
E	Soil	*Chryseobacterium* sp. (6)	9.15 × 10^–4^ – 2.43 × 10^–3^
		*Curtobacterium* sp. (1)	2.64 × 10^–4^
		*Dyadobacter* sp. (1)	1.53 × 10^–3^
		*Lysobacter* sp. (1)	8.85 × 10^–4^
		*Microbacterium* sp. (1)	1.75 × 10^–4^
		*Sphingobacterium* sp. (3)	7.20 × 10^–4^ – 1.69 × 10^–3^
		*Stenotrophomonas* sp. (14)	2.28 × 10^–4^ – 3.66 × 10^–3^
		*Variovorax* sp. (1)	1.44 × 10^–3^
	Vegetable	*Chryseobacterium* sp. (1)	1.14 × 10^–3^
		*Stenotrophomonas* sp. (1)	1.33 × 10^–3^
G	Soil	*Chryseobacterium* sp. (1)	8.40 × 10^–4^
	Vegetable	*Chryseobacterium* sp. (1)	1.87 × 10^–3^
		*Sphingobacterium* sp. (1)	1.81 × 10^–3^
		*Stenotrophomonas* sp. (1)	1.53 × 10^–3^
O	Soil	*Pseudomonas* sp. (1)	1.44 × 10^–3^
		*Stenotrophomonas* sp. (1)	2.43 × 10^–3^
	Vegetable	*Chryseobacterium* sp. (1)	1.60 × 10^–3^
		*Stenotrophomonas* sp. (3)	8.80 × 10^–4^ – 1.75 × 10^–3^

### Antimicrobial Resistance Genes Revealed by Whole-Genome Sequencing

A number of ARGs were detected in the sequenced isolates, with multidrug efflux genes being most common (Supplementary Table 2). *Acinetobacter calcoaceticus* carried genes related to multidrug efflux pump AdeIJK conferring resistance to β-lactams, chloramphenicol, erythromycin, fluoroquinolones, lincosamides, and tetracycline. Multidrug *mex* efflux genes (*mex*B, *mex*F, *mex*K, and *mex*W) were detected in *Lysobacter gummosus* and *Pseudomonas atacamensis.* SmeDEF is a multidrug efflux pump for resistance to quinolones, tetracyclines, macrolides, chloramphenicol, and novobiocin in *Stenotrophomonas maltophilia*. The three genes, *sme*D, *sme*E, and *sme*F, were detected in four *Stenotrophomonas* isolates that also had *oqx*B, an efflux pump conferring quinolone resistance. High MIC to multiple antibiotics, including ciprofloxacin, was observed in these isolates. *emr*B is an efflux pump gene targeting fluoroquinolone antibiotics. Both *emr*B and *oqx*B were detected in two *Pantoea*, one of which had high MIC to nalidixic acid whereas the other showed low MIC to all antibiotics, suggesting pan-susceptibility. *cat* encoding chloramphenicol resistance was detected in three *Agrobacterium*. Multidrug resistance gene *crp* was detected in *Rahnella* and *Pantoea*, and so was tetracycline resistance gene *tet*42 identified in two *Microbacterium*. Resistance genes to additional antibiotics such as β-lactam (*cgb-1*), landomycin (*lnd-4*), rifampin (rifampin resistance gene), and vancomycin (*van*RO) were also detected but at a lower frequency.

Antimicrobial resistance gene identity did not always agree with antimicrobial susceptibility profiles. For example, despite the presence of *crp, emr*B, and *oqx*B, *Pantoea* sp. (OVA07A) appeared to be pan-susceptible with low MICs to all tested antibiotics. On the other hand, no ARG was detected in *Sphingobacterium* GVS05A with high MICs to ampicillin, cefoxitin, chloramphenicol, gentamicin, and nalidixic acid. The same situation applied to one *Chryseobacterium* (OSA05B) and two *Lysinibacillus* (EVS05B and OSS05C) that demonstrated high MICs to multiple antibiotics but with no ARGs identified. *Microbacterium* (EST19A) carried a rifampin resistance gene; however, broth microdilution failed to detect a high MIC based on the rifampin resistance breakpoint of *S. aureus*.

## Discussion

Despite extensive research on soil microorganisms in agricultural environment as a reservoir of antimicrobial resistance, it remains largely unexplored as to the prevalence of antimicrobial-resistant soil microorganisms in urban agriculture as well as their potential of transferring antimicrobial resistance to bacteria of food safety significance. In this study, a diverse collection of soil bacteria recovered from Detroit urban community gardens demonstrated a high MIC to a wide range of antibiotics, including those of clinical significance. ARGs commonly detected in clinical bacteria were found in soil isolates as well, suggesting a shared pool of ARG between clinical and environmental microorganisms. Added to the significance was the successful conjugation of antimicrobial resistance to a susceptible *E. coli* or *Enterococcus faecalis* recipient, underlying that soil bacteria could transfer antimicrobial resistance to foodborne pathogens and thus pose a public health concern.

The phyla and genera identified in this study were aligned with common soil bacteria (Janssen, 2006). The prevalence of *Proteobacteria*, *Bacteroidetes*, *Actinobacteria*, and *Firmicutes* is in agreement with a previous study on six farming fields (Armalytë et al., 2019). In addition, soils collected from different ecosystems, including tropical forest/grassland, boreal forest/tundra, humid temperate forest, humid temperate grassland, dry forest, and dry grassland/shrubland across North America also reported *Actinobacteria*, *Acidobacteria*, *Bacteroidetes*, α- *Proteobacteria*, β- *Proteobacteria*, and *Firmicutes* as the dominant bacterial phyla (Fierer et al., 2007).

The predominance of *Proteobacteria* was demonstrated by both the cell culturing and high-throughput 16S rRNA sequencing methods. A previous research reported 37.6% of *Proteobacteria* in maize-root and bulk soils (Aguirre-von-Wobeser et al., 2018), which corroborates the current study (35.94 – 38.11%). The top four phyla have also been reported in forest soils (Wei et al., 2017), urban community garden soils (Mafiz et al., 2018), and agricultural soils (Dube et al., 2019). The discrepancy between the two methods in the ranking of the phyla excluding *Proteobacteria* can be explained by the culturability of bacteria because culturable bacteria represent only 1% of total bacterial diversity in soil (Schloss and Handelsman, 2003; Demanèche et al., 2008), whereas the sequencing method captures both culturable and non-culturable microorganisms (Handelsman, 2004).

Since no standard resistance breakpoints are available for soil bacteria, we were unable to interpret the antimicrobial susceptibility status of the bacteria. However, the high MIC demonstrated by soil bacteria according to the breakpoints of *E. coli* and *S. aureus* in this study can be an indication of potential antimicrobial resistance. This interpretation is supported by one of our previous research where ampicillin-resistant *E. coli* were recovered from urban community gardens as well as *Enterococcus* resistant to ampicillin, ciprofloxacin, erythromycin, streptomycin, and tetracycline (Perera et al., 2020). Another study reported over 80% of bacteria in agriculture, urban, and pristine soils resistant to 16 – 23 antibiotics of all known classes (Walsh and Duffy, 2013). This collectively suggests the extent of antimicrobial resistance reservoir associated with agricultural environment. The skewness toward high MICs in Gram-negative bacteria compared to Gram-positive bacteria in this study suggest a higher potential of Gram-negative bacteria to be antimicrobial resistant, which can be explained by their extra outer membrane that provides additional protection against antibiotics in the environment as well as those used during microbial isolation (Delcour, 2009). This also supports the predominance of Gram-negative bacteria in the recovered microorganisms because of their better survival during the isolation process.

The successful transfer of tetracycline resistance from soil bacteria to *E. coli* or *Enterococcus faecalis* JH2-2 via conjugation indicates the potential of environmental bacteria to spread antimicrobial resistance to pathogenic bacteria. This raised a food safety concern because both recipients are frequently found in the food supply (Holvoet et al., 2013; Micallef et al., 2013; Araújo et al., 2017). Bacteria of both soil and vegetable origins were capable of tetracycline resistance transfer with similar conjugation frequencies, suggesting that soil bacteria in the food production environment, regardless of source of isolation, are all potential contributors of antimicrobial resistance in foodborne bacteria.

The detection of efflux pump genes in soil bacteria may explain the prevalence of bacteria with high MICs. This echoes a previous report where efflux was the prime mechanism for multidrug resistance in bacteria recovered from soil (Walsh and Duffy, 2013). Another research also demonstrated multidrug efflux pumps in *E. coli* from urban gardens regardless of their antimicrobial susceptibility phenotypes (Perera et al., 2020). *Acinetobacter calcoaceticus* carried *ade*I, *ade*J, and *ade*K, raising a public health concern because the AdeIJK pump confers resistance to multiple antibiotics of clinical importance, including β-lactams, chloramphenicol, erythromycin, fluoroquinolones, fusidic acid, lincosamides, novobiocin, rifampin, tetracycline, and trimethoprim (Damier-Piolle et al., 2008). The same genes have also been detected in an emerging clinical pathogen, *Acinetobacter baumannii* (Kor et al., 2014). Future research should investigate the specificity of the AdeIJK pump genes in *Acinetobacter* as well as the virulence potential of soil-originating *Acinetobacter spp.* The presence of *sme*DEF in the four *Stenotrophomonas* isolates may have contributed to the high MIC to chloramphenicol, ciprofloxacin, and tetracyclines. The high MIC to ciprofloxacin was also consistent with the detection of *oqx*B, a quinolone resistance efflux pump gene, in the same isolates.

The high MIC to antibiotics did not always show a genetic basis in the isolates. This is evidenced by *Chryseobacterium, Sphingobacterium*, and *Lysinibacillus* demonstrating high MIC to multiple antibiotics yet no ARG detected. The discrepancy could be due to the presence of novel or unidentified ARGs. Also, the gene searching was based on the most stringent criterion in this study, which may have excluded sequences that did not show exact matches. Lowering the search stringency may reveal more potential ARG variants. On the contrary, ARGs identified in the isolates may not always lead to a high MIC to the corresponding antibiotic. For example, a *Pantoea* (OVA07A) carrying *oqxB, emrB*, and *crp* failed to show a high MIC to any antibiotics tested. *rphB* conferring rifampin resistance was detected in *Neobacillus bataviensis* yet a high MIC to rifampin was not observed. This phenotype-genotype inconsistency could be from a failed gene expression, although phenotypic interpretation could be another factor due to the lack of standard breakpoints established for environmental isolates. Previous research has used either 20 μg/ml (D’Costa et al., 2006; Walsh and Duffy, 2013) or clinical breakpoints (Popowska et al., 2012; Armalytë et al., 2019) to define antimicrobial resistance for environmental bacteria. This could cause misinterpretation of the environmental data. Consequently, there is a need to develop interpretation criteria for these isolates to better understand the public health risk of environmental bacteria.

In conclusion, this study demonstrated that urban community gardens could serve as a potential source of antimicrobial resistance that may transfer to bacteria of food safety concern. Environmental and clinical bacteria share a common pool of ARGs, suggesting that genetic exchange at the human environment interface should be considered when developing antimicrobial resistance control. Future research should include expanding the environmental database of ARGs and antimicrobial resistance phenotypes to enhance our capability to interpret the public health implication of antimicrobial resistance in the environment.

## Data Availability Statement

The datasets presented in this study can be found in online repositories. Genome sequence data have been deposited at NCBI under BioProject PRJNA749625. GenBank BioSample accession numbers are included in Supplementary Table 1.

## Author Contributions

AM and YH performed the experiment. YZ and WZ designed the experiment. AM and YZ wrote the manuscript. YZ, YH, and WZ revised the manuscript. All authors contributed to the article and approved the submitted version.

## Conflict of Interest

The authors declare that the research was conducted in the absence of any commercial or financial relationships that could be construed as a potential conflict of interest.

## Publisher’s Note

All claims expressed in this article are solely those of the authors and do not necessarily represent those of their affiliated organizations, or those of the publisher, the editors and the reviewers. Any product that may be evaluated in this article, or claim that may be made by its manufacturer, is not guaranteed or endorsed by the publisher.

## Footnotes

1
http://rdp.cme.msu.edu/misc/about.jsp

2
https://www.bioinformatics.babraham.ac.uk/projects/fastqc/

3
www.usadellab.org/cms/?page=trimmomatic

4
http://cab.spbu.ru/software/spades/

5
https://card.mcmaster.ca/
