# Supplementary material for: Soil Bacteria in Urban Community Gardens Have the Potential to Disseminate Antimicrobial Resistance Through Horizontal Gene Transfer
Source: Front Microbiol. 2021 Nov 23;12:771707. doi: 10.3389/fmicb.2021.771707 (PMC8650581; doi:10.3389/fmicb.2021.771707)
Supplement: Supplementary file 1 [file Data_Sheet_1.zip › Supplementary Document S1.PDF]

Supplementary Document S1. Agricultural Practices on the Garden Sites

Garden E: The site had been an abandoned lot before being developed as a vegetable garden since 2000. The soil is original to the site. When it was first developed the site was cleared of brick, concrete and other debris. This was moved to make the berm that surrounds the site. When it was first started two tractor trailer loads of compost was brought in, but since that time all compost has been made on site from pre-consumer food scrapes, and yard waste (leaves, grass clippings etc). The garden uses municipal and rainwater for irrigation, which is common in many urban community gardens in the metro Detroit area. A variety of root vegetables and leafy greens were grown, such as potatoes, squash, onions, garlic, and leek etc.

Garden G: Topsoil of 2-3 foot deep from a nearby playground was brought to the site in 2005. Mineral fertilizer and onsite plant compost were applied. Root and leafy vegetables were grown, including turnips, scallions, collard greens, and mustard.

Garden O: The site is based in part of one of the historic chicken coops, formerly used as a Day Care Center following its decommission as a working farm in 1957. Soils were amended with plant meals (alfalfa meals) and minerals (Azomite, a naturally rich soil re-mineralizer for plants). Root and leafy vegetables such as potatoes, carrots, collard greens, and kales were grown.
