# Supplementary material for: Soil Bacteria in Urban Community Gardens Have the Potential to Disseminate Antimicrobial Resistance Through Horizontal Gene Transfer
Source: Front Microbiol. 2021 Nov 23;12:771707. doi: 10.3389/fmicb.2021.771707 (PMC8650581; doi:10.3389/fmicb.2021.771707)
Supplement: Supplementary file 1 [file Data_Sheet_1.zip › Table S1.PDF]

**Table S1** Genomic Assembly Quality Data of the Isolates

| ID | Bacteria Name | Genome Size | No. of ambiguous bases | No. of contigs | Largest contig | Total length | Mean contig length | N50 | N75 | L50 | L75 | Biosample accession no. |
|---|---|---|---|---|---|---|---|---|---|---|---|---|
| OVA10B | *Acinetobacter calcoaceticus* | 4021045 | 8 | 592 | 1288884 | 4318045 | 7294 | 490068 | 289894 | 3 | 5 | SAMN20394460 |
| GVS04A | *Agrobacterium tumefaciens* | 5793907 | 0 | 59 | 718701 | 5673969 | 96169 | 338905 | 177535 | 6 | 11 | SAMN20394472 |
| GVS01A | *Agrobacterium tumefaciens* | 5415768 | 0 | 34 | 1783709 | 5275475 | 155161 | 861394 | 371967 | 2 | 5 | SAMN20394470 |
| EVA06B | *Agrobacterium tumefaciens* | 5408651 | 0 | 24 | 2417221 | 5297783 | 220741 | 840693 | 380730 | 2 | 4 | SAMN20394471 |
| EST11A | *Chryseobacterium lathyri* | 4796907 | 9 | 12 | 1579788 | 4503710 | 375309 | 1266256 | 1149636 | 2 | 3 | SAMN20394461 |
| OSA05B | *Chryseobacterium* sp. | 5298154 | 8 | 87 | 2560750 | 5225823 | 60067 | 1059831 | 909680 | 2 | 3 | SAMN20394462 |
| GVT01B | *Chryseobacterium* sp. | 5353077 | 10 | 18 | 1273353 | 5143328 | 285740 | 726078 | 547171 | 3 | 5 | SAMN20394463 |
| EVS05B | *Lysinibacillus fusiformis* | 4738547 | 8 | 546 | 1593239 | 4977525 | 9116 | 1096220 | 208818 | 2 | 6 | SAMN20394480 |
| OSS05C | *Lysinibacillus sphaericus* | 5421625 | 41 | 70 | 338044 | 4799397 | 68563 | 162443 | 111517 | 11 | 21 | SAMN20394481 |
| EVS02B | *Lysobacter gummosus* | 6268123 | 98 | 63 | 635243 | 6246389 | 99149 | 155245 | 109439 | 13 | 24 | SAMN20394464 |
| ESA13C | *Lysobacter* sp. | 6205434 | 116 | 43 | 950200 | 5814535 | 135222 | 363690 | 248820 | 4 | 9 | SAMN20394465 |
| OVT16B | *Microbacterium* sp. | 3999223 | 19 | 22 | 3885675 | 3897143 | 177143 | 3885675 | 3885675 | 1 | 1 | SAMN20394483 |
| EST19A | *Microbacterium* sp. | 4605771 | 99 | 9 | 2014193 | 4445517 | 493946 | 1608272 | 1608272 | 2 | 2 | SAMN20394482 |
| GVA02B | *Neobacillus bataviensis* | 7129380 | 0 | 55 | 1007127 | 6300449 | 114554 | 491863 | 220738 | 5 | 10 | SAMN20394479 |
| OVA06A | *Pantoea agglomerans* | 5032067 | 0 | 37 | 1488627 | 4848188 | 131032 | 580016 | 450324 | 3 | 5 | SAMN20394466 |
| OVA07A | *Pantoea* sp. | 4955614 | 22 | 20 | 2326846 | 4780402 | 239020 | 584181 | 464927 | 2 | 4 | SAMN20394467 |
| GVA01A | *Pseudomonas atacamensis* | 6318431 | 190 | 20 | 1488007 | 5874823 | 293741 | 638311 | 375320 | 4 | 6 | SAMN20394468 |
| GSA61A | *Rahnella* sp. | 5829506 | 0 | 61 | 553562 | 5619763 | 92127 | 268314 | 109534 | 7 | 15 | SAMN20394469 |
| GVS05A | *Sphingobacterium* sp. | 6535891 | 0 | 21 | 1141412 | 6287888 | 299423 | 659634 | 377223 | 4 | 8 | SAMN20394474 |
| OVT16A | *Stenotrophomonas indicatrix* | 5166508 | 197 | 11 | 3673989 | 4600420 | 418220 | 3673989 | 3673989 | 1 | 1 | SAMN20394476 |
| GST33B | *Stenotrophomonas indicatrix* | 4762338 | 9 | 12 | 2513045 | 4597257 | 383105 | 2513045 | 1137476 | 1 | 2 | SAMN20394477 |
| ESA45A | *Stenotrophomonas maltophilia* | 4784051 | 104 | 18 | 1625717 | 4524418 | 251357 | 489395 | 415431 | 3 | 5 | SAMN20394475 |
| OVS01A | *Stenotrophomonas* sp. | 4838535 | 17 | 21 | 1829688 | 4549116 | 216625 | 804334 | 267642 | 2 | 5 | SAMN20394478 |
