# Supplementary material for: Soil Bacteria in Urban Community Gardens Have the Potential to Disseminate Antimicrobial Resistance Through Horizontal Gene Transfer
Source: Front Microbiol. 2021 Nov 23;12:771707. doi: 10.3389/fmicb.2021.771707 (PMC8650581; doi:10.3389/fmicb.2021.771707)
Supplement: Supplementary file 1 [file Data_Sheet_1.zip › Table S2.PDF]

Table S2. Antibiotic Resistance Genes Identified by Whole-Genome Sequencing in the Isolates

Bacteria ID	Bacteria	Source	Identified Antibiotic Resistance Genes (ARGs)	Phenotypes
OVA10B	*Acinetobacter calcoaceticus*	Vegetable	*abe*M, *abe*S, *adc*, *ade*G, *ade*H, *ade*I *ade*J, *ade*K, *oxa*	AMP, AUG2, AXO, CHL, FOX, GEN
EST11A	*Chryseobacterium lathyri*	Soil	*Ind*-4	AMP, AUG2, AXO, CHL, FOX, GEN, NAL, TET
OSA05B	*Chryseobacterium* sp.	Soil	None	AMP, AUG2, AXO, CIP, FOX, GEN, NAL, TET
GVT01B	*Chryseobacterium* sp.	Vegetable	*cgb*-1 b-lactamase	AMP, AUG2, AXO, CIP, CHL, FOX, GEN, TET
EVS02B	*Lysobacter gummosus*	Vegetable	*mex*K, *sme*E	AMP, AXO
ESA13C	*Lysobacter* sp.	Soil	*sme*E	AMP, AUG2, AXO, CHL, CIP, FOX, TET,
OVA06A	*Pantoea agglomerans*	Vegetable	*crp*, *emr*B, *oqx*B	AMP, AUG2, AXO, CHL, FOX, GEN, NAL,
OVA07A	*Pantoea* sp.	Vegetable	*crp*, *emr*B, *oqx*B	NONE
GVA01A	*Pseudomonas atacamensis*	Vegetable	*mex*B, *mex*F, *mex*K, *mex*W,	AMP, AUG2, AXO, CHL, FOX, NAL
GSA61A	*Rahnella* sp.	Soil	*crp*	AMP, AUG2, AXO, FOX, NAL, TET
GVS01A	*Agrobacterium tumefaciens*	Vegetable	*cat*	AMP, AUG2, CHL, FOX, NAL
EVA06B	*Agrobacterium tumefaciens*	Vegetable	*cat*	CHL, FOX
GVS04A	*Agrobacterium tumefaciens*	Vegetable	*cat*	AMP, CHL, NAL
GVS05A	*Sphingobacterium* sp.	Vegetable	None	AMP, CHL, FOX, GEN, NAL
ESA45A	*Stenotrophomonas maltophilia*	Soil	*oqx*B, *sme*D, *sme*E, *sme*F	AMP, AUG2, AXO, CHL, CIP, FOX, GEN, NAL, TET
OVT16A	*Stenotrophomonas indicatrix*	Vegetable	*oqx*B, *sme*D, *sme*E, *sme*F,	AMP, AUG2, AXO, CHL, CIP, FOX, GEN, TET
GST33B	*Stenotrophomonas indicatrix*	Soil	*oqx*B, *sme*D, *sme*E, *sme*F	AMP, AUG2, AXO, CHL, CIP, FOX, GEN, NAL, TET
OVS01A	*Stenotrophomonas* sp.	Vegetable	*oqx*B, *sme*D, *sme*E, *sme*F	AMP, AUG2, AXO, CHL, CIP, FOX, GEN, TET
GVA02B	*Neobacillus bataviensis*	Vegetable	*rph*B	GEN, PEN
EVS05B	*Lysinibacillus fusiformis*	Vegetable	None	CIP, ERY, GEN, KAN, PEN
OSS05C	*Lysinibacillus sphaericus*	Soil	None	GEN, KAN, PEN
EST19A	*Microbacterium* sp.	Soil	rifampin resistance, *tet*42	CIP, DAP, ERY, GEN, KAN, LIN, NIT, PEN, SYN, TET
OVT16B	*Microbacterium* sp.	Vegetable	*tet*42, *van*RO	CIP, CHL, ERY, GEN, KAN, PEN, SYN, TET

Note: AMP=Ampicillin, AUG2=Amoxicillin / clavulanic acid 2:1 ratio, AXO=Ceftriaxone, CIP=Ciprofloxacin, CHL=Chloramphenicol, FOX=Cefoxitin, GEN=Gentamicin, KAN=Kanamycin, NAL=Nalidixic Acid, PEN=Penicillin, SYN=Quinupristin / dalfopristin, TET=Tetracycline
